# Insulin resistance and atrial fibrillation: from disease onset to post-ablation outcomes: a systematic review and meta-analysis

**DOI:** 10.3389/fcvm.2025.1700730

**Published:** 2026-01-08

**Authors:** Tao Huang, Ze Fang, Qiongfang Zhang, Yanmei Li, Lili Shi

**Affiliations:** Zhongjiang County People’s Hospital, Deyang, Sichuan, China

**Keywords:** ablation, atrial fibrillation, early identification, insulin resistance, mechanism, metabolic syndrome, personalized management, recurrence

## Abstract

**Objective:**

To systematically evaluate the impact of insulin resistance (IR) on the risk of new-onset atrial fibrillation (AF) and post-ablation recurrence, and to elucidate underlying mechanisms and translational implications for early identification and precision management of high-risk populations.

**Methods:**

A comprehensive meta-analysis was conducted by integrating 30 high-quality cohort studies from multiple countries, incorporating IR markers such as TyG index and HOMA-IR. Random-effects models were used to estimate the association between IR and both AF incidence and ablation recurrence. Subgroup analyses, sensitivity analyses, meta-regression, and GRADE quality assessment were performed to explore heterogeneity and robustness. Mechanistic pathways were systematically summarized. This study was registered in PROSPERO (CRD420251142441).

**Results:**

Pooled results demonstrated that IR significantly increased the risk of new-onset AF (HR = 1.34, 95%CI: 1.24–1.46) and post-ablation recurrence (HR = 1.57, 95%CI: 1.39–1.78), with consistent effects across subgroups by country, IR index, study type, and follow-up. Mechanistic evidence revealed that IR promotes AF development and recurrence via enhanced inflammation, oxidative stress, atrial structural remodeling, and electrical abnormalities. IR-related indices showed higher clinical utility for risk stratification in Chinese and Asian populations. Meta-regression and sensitivity analyses confirmed the robustness of these findings. The quality of evidence was rated as moderate.

**Conclusion:**

Insulin resistance is an independent risk factor for both new-onset and recurrent AF. IR-related indices have translational value for early risk identification and personalized management. Incorporating IR assessment into AF management and prevention strategies—integrating metabolic and electrophysiological perspectives—may optimize long-term outcomes and provide a foundation for precision medicine and tailored follow-up in AF care.

**Systematic Review Registration:**

https://www.crd.york.ac.uk/prospero/display_record.php?ID=CRD420251142441, PROSPERO CRD420251142441.

## 1 Introduction

### 1.1 Background

Atrial fibrillation (AF) is the most common persistent cardiac arrhythmia worldwide. Its incidence increases with age and has become one of the main causes of stroke, heart failure, and cardiovascular death (1–3). Epidemiological data show that the number of AF patients worldwide is continuously increasing, and it is estimated that by 2050, the number of AF patients in the United States will reach 6–12 million, and 17.9 million in Europe (1). In China, with the changing spectrum of cardiovascular diseases and an aging population, the incidence of AF is also increasing. Epidemiological surveys indicate that the prevalence of AF among Chinese adults aged 35 and above is about 0.71%, and as high as 1.83% among the elderly over 60 years old, with the number of patients exceeding 8 million, placing tremendous pressure on the medical system and the social economy (4). AF not only increases the risk of stroke and heart failure but also significantly elevates all-cause mortality and disability rates (2–4). Therefore, in-depth exploration of the pathogenesis and risk factors of AF is of great significance for reducing the burden of cardiovascular events.

Insulin resistance (IR) is the core mechanism of chronic metabolic diseases such as type 2 diabetes, obesity, metabolic syndrome, and nonalcoholic fatty liver disease, and is also the common basis for the increasing global burden of chronic diseases. IR not only promotes the progression of type 2 diabetes and atherosclerosis, but can also, through mechanisms such as inflammatory activation (e.g., elevated factors like TNF-α, IL-6), oxidative stress, endothelial dysfunction, lipotoxicity, and neurohumoral imbalance, lead to cardiac structural and electrophysiological remodeling (including atrial fibrosis, collagen deposition, cardiomyocyte apoptosis, ion channel remodeling, etc.), thereby increasing the risk of atrial fibrillation (1, 2, 4, 5).

Multiple large-scale prospective cohort studies and meta-analyses have shown that insulin resistance and its related diseases (such as type 2 diabetes, metabolic syndrome, and nonalcoholic fatty liver disease) all significantly increase the risk of atrial fibrillation (1–4). Meta-analyses indicate that diabetes can increase the risk of atrial fibrillation by 23%–49%, and the risk is even higher in women, younger patients, and those with high HbA1c levels (2–4). IR is not only a risk factor for the occurrence of atrial fibrillation but also has an important impact on the outcomes after catheter ablation of atrial fibrillation. Related studies have shown that patients with elevated IR levels have more significant atrial remodeling and fibrosis after ablation, poorer electrophysiological stability, a significantly increased risk of recurrence, and worse long-term prognosis (4–6).

At present, direct quantitative evidence regarding the relationship between IR and the occurrence of atrial fibrillation and post-ablation outcomes is still relatively limited. Most studies focus on type 2 diabetes as the entry point and lack high-quality meta-analyses that systematically review the “dual outcomes” of atrial fibrillation with IR as the core (1–3, 6). This study takes insulin resistance as the core variable, systematically incorporates the latest cohort data from China and abroad, comprehensively evaluates the independent effects of IR on the two major endpoints of new-onset and recurrent atrial fibrillation after ablation, and reviews the mechanisms such as inflammation, structural remodeling, and electrophysiological abnormalities. Evidence from large Chinese and Asian cohorts has been added to provide new support for the application value of IR indicators in regional risk stratification and individualized management. The results of this study help to improve the early prevention strategies for high-risk populations in China and globally.

### 1.2 Objective of the study

This study aims to systematically evaluate the impact of insulin resistance on the risk of new-onset atrial fibrillation and recurrence after ablation, and to clarify its clinical significance as an independent risk factor. By integrating high-quality cohort data from both domestic and international sources, this study comprehensively analyzes the association and mechanisms between IR and the “dual outcomes” of atrial fibrillation, and further evaluates the stratified predictive value of IR-related indicators in different populations. The study strives to provide evidence-based support for the early identification, individualized management, and targeted IR intervention for high-risk populations of atrial fibrillation, and to promote the practical optimization of AF prevention and control strategies.

## 2 Methods

### 2.1 Search strategy

This study was designed in strict accordance with the PRISMA statement and has been registered in PROSPERO (Registration Number: CRD420251142441). Major databases such as PubMed, Embase, and Web of Science were systematically searched, with the search period ending on August 24, 2025. There were no restrictions on language or publication date. The search strategy combined subject headings and free-text terms, covering relevant topics such as “insulin resistance,” “atrial fibrillation,” “ablation,” “recurrence,” and “prognosis.” The specific search formulas and results are shown in Supplementary Table 1. Additionally, supplementary searches were conducted through the references of included studies and related reviews to ensure the comprehensiveness and authority of the literature collection.

### 2.2 Inclusion and exclusion criteria

Inclusion criteria:

(1) The study subjects were adults (≥18 years old);
(2) The original study was a cohort study, case-control study, or randomized controlled trial, and reported the relationship between insulin resistance (IR)-related indicators and the risk of atrial fibrillation occurrence or post-ablation outcomes (such as recurrence, prognosis, etc.);
(3) Relative risk (RR), hazard ratio (HR), odds ratio (OR) and their 95% confidence intervals were clearly provided or could be calculated, or raw data were available for estimation;
(4) If there were multiple reports of the same population, only the study with the most complete data and the longest follow-up was included.

Exclusion criteria:

(1) Animal experiments, *in vitro* studies, reviews, meta-analyses, case reports, commentaries, abstracts, letters, and other non-original research articles;
(2) Studies that did not provide data on the correlation between insulin resistance-related indicators and atrial fibrillation outcomes, or from which effect values and confidence intervals could not be extracted;
(3) Studies that included only special populations (such as children, pregnant women, etc.) or those with other serious underlying diseases affecting the interpretation of results;
(4) Full text not available or data incomplete and unable to be supplemented.

### 2.3 Data extraction

Data extraction was performed by five researchers using a double-independent and cross-checked approach. The literature was independently screened and data were extracted, including: first author, year of publication, study type, country (region), sample size, mean age and gender ratio, method of insulin resistance assessment, type of atrial fibrillation ablation, follow-up duration, main outcome indicators (incidence of atrial fibrillation and recurrence after ablation), effect estimates (RR, HR, OR and their 95% confidence intervals), and major confounding factors adjusted for.

Information on ablation modality, definition of recurrence, and follow-up protocol was extracted where available. Due to incomplete and inconsistent reporting of these variables across studies, further stratified analyses based on ablation-related characteristics were limited.

In case of disagreement during the extraction process, a third researcher was consulted for adjudication; if consensus could not be reached, all five researchers discussed and made a collective decision.

### 2.4 Quality assessment

All the studies included in this research were cohort studies, and the ROBINS-I (Risk Of Bias In Non-randomized Studies of Interventions) tool was used to assess the risk of bias. Two researchers independently evaluated each study across seven domains (including confounding bias, selection bias, classification bias, deviation bias, measurement bias, missing data bias, and reporting bias) and made an overall judgment on the risk level of bias. In case of disagreement, all researchers participated in the discussion and made the final decision.

### 2.5 Statistical analysis

All included studies were managed and deduplicated using Zotero literature management software. Data analysis was performed using R language and relevant meta-analysis packages. For combined binary variables, the risk ratio and its 95% confidence interval were used as effect measures, and a random effects model was selected according to the actual data. Heterogeneity was assessed by the I² statistic and Q test. When significant heterogeneity existed, subgroup analysis and sensitivity analysis were further performed to explore potential sources. For outcomes influenced by multiple factors or with confounding variables, meta-regression analysis was used to investigate the impact of covariates on the results. Publication bias was assessed by funnel plot and Egger's test.

## 3 Results

### 3.1 Literature screening process

**Figure 1:**
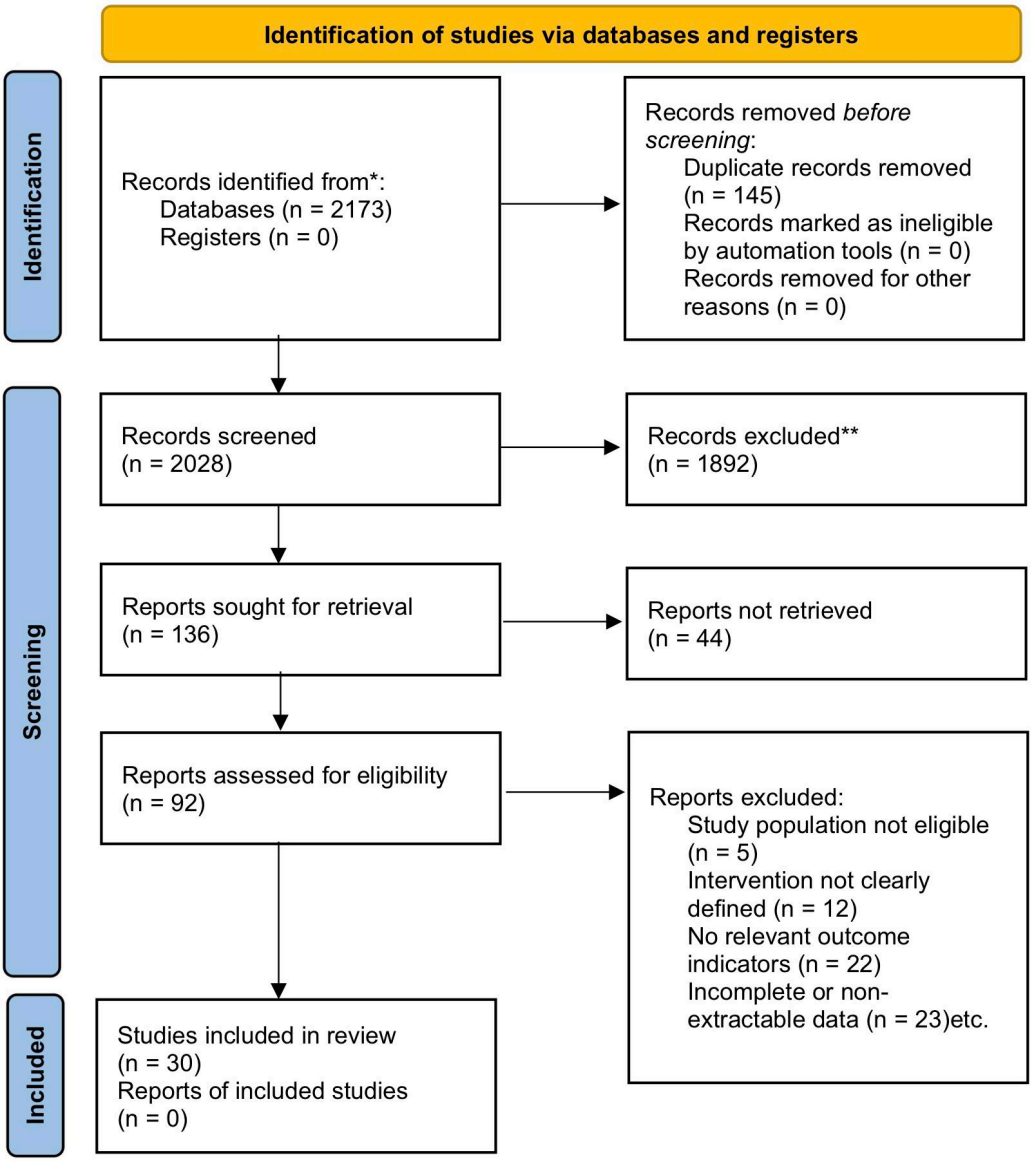
PRISMA flow chart.

A total of 2,173 articles were retrieved in this study. After automatic and manual deduplication, 2,028 articles remained. After initial screening of titles and abstracts, 1,892 articles that clearly did not meet the criteria were excluded. The remaining 136 articles were retrieved for full-text evaluation, of which 44 were excluded due to unavailability of full text. After rigorous full-text screening of the remaining 92 articles, a total of 30 studies meeting the criteria were finally included. The main reasons for exclusion included: study subjects not meeting the criteria (*n* = 5), intervention measures not clearly defined (*n* = 12), relevant outcome indicators not reported (*n* = 22), and incomplete or non-extractable data (*n* = 23). The specific screening process is shown in the PRISMA flow chart (Figure 1).

### 3.2 Basic characteristics of included studies

A total of 30 cohort studies were included in this study, comprising retrospective cohorts (RCS) and prospective cohorts (PCS), involving seven countries and regions including China, the United States, the United Kingdom, Japan, South Korea, Spain, and Sweden. The sample sizes of the studies varied greatly, ranging from a minimum of 114 cases to a maximum of 409,705 cases. The average age of the included populations was mostly over 50 years old, and the proportion of males ranged from 41.9% to 80.2%. All studies used objective indicators to assess insulin resistance status, including three commonly used methods: TyG index, HOMA-IR, and eGDR. Among them, TyG index and HOMA-IR were the most widely used, with some studies using eGDR for supplementary analysis.The follow-up duration of the cohorts ranged from 0.8 to 24.3 years. Most studies reported the impact of insulin resistance on the risk of atrial fibrillation, while some focused on recurrence and clinical prognosis after catheter ablation for atrial fibrillation. The main characteristics of the included studies are shown in Table 1, and detailed information can be found in Supplementary Table 2.

**Table 1:** Characteristics of included studies.

First author	Year	Study design	Sample Size	Male %	Mean age	IR index	Follow-up (yr)	AF incidence risk (HR, 95%CI)	Post-ablation outcome (HR, 95% CI)
Yongwei Huang	2024	RCS	1,707	55.42%	68.00	TyG index	1 (yr)	1.55 (1.31–2.01)	NA
Zhe Wang	2024	RCS	2,242	63.38%	60.74	TyG index	1 (yr)	NA	1.71 (1.41–2.07)
Zhihong Zuo	2025	RCS	4,276	59.71%	63.24	TyG index	1 (yr)	1.58 (1.23–2.02)	NA
Sixiang Jia	2024	RCS	997	63.19%	63.21	TyG index	1–3 (yr)	NA	1.26 (1.09–1.45)
Xiao Liu	2023	PCS	11,851	44.40%	54.00	TyG index	24.3 (yr)	1.18 (1.03–1.37)	NA
Yan Luo	2024	RCS	910	49.60%	65.98	TyG index	1–1.2 (yr)	NA	1.47 (1.16–1.87)
Caravaca	2025	PCS	2,902	47.50%	77.00	TyG index	2 (yr)	NA	1.82 (1.15–2.89)
Aobo Gong	2025	RCS	864	55.30%	67.69	TyG index	3.9 (yr)	NA	1.77 (1.44–2.17)
Yonggu Lee	2020	PCS	8,175	46.70%	51.50	HOMA-IR	11.5(yr)	1.61 (1.14–2.29)	NA
Yang Ling	2022	RCS	549	80.20%	63.00	TyG index	2.9 (yr)	1.58 (1.27–1.97)	NA
Hao Huang	2025	PCS	360,953	45.10%	56.30	eGDR	13.8 (yr)	1.18 (0.78–1.58)	NA
Shanshan Shi	2024	PCS	409,705	45.70%	56.40	TyG index	13.9 (yr)	1.32 (1.17–1.87)	NA
Fontes	2012	PCS	3,023	45.20%	59.20	HOMA-IR	10 (yr)	1.18 (0.84–1.65)	NA
Tang	2022	RCS	275	69.40%	57.30	TyG index	2.2 (yr)	NA	2.02 (1.41–4.12)
Li XZ	2024	RCS	899	41.90%	64.45	eGDR	0.9 (yr)	NA	1.08 (0.5–1.87)
Kan	2024	RCS	325	60.90%	60.60	TyG index	1 (yr)	NA	3.39 (2.08–5.53)
Johnson	2015	RCS	1,414	77.00%	62.00	HOMA-IR	0.8–3.2 (yr)	NA	1.57 (1.08–2.28)
Tze-Fan Chao	2013	RCS	122,524	73.70%	50.60	HOMA-IR	6.3 (yr)	1.19 (1.01–1.59)	NA
Qing Yan	2024	RCS	375	64.30%	63.20	TyG index	1 (yr)	NA	1.45 (0.78–3.78)
Naoko Hijioka	2018	PCS	114	78.00%	62.60	HOMA-IR	1 (yr)	NA	1.29 (1.08–1.53)
Jingwei Zhang	2023	RCS	424	70.80%	58.20	TyG index	2.8 (yr)	NA	2.02 (1.37–3.25)
Zhen Tan	2025	PCS	31,733	57.28%	59.19	eGDR	12.8 (yr)	1.54 (1.31–1.76)	NA
Aiko Takami	2025	RCS	818	70.70%	67.00	HOMA-IR	2 (yr)	NA	2.62 (1.44–4.80)
Pil-Sung Yang	2016	PCS	142	78.20%	63.30	HOMA-IR	0.8–3.2 (yr)	NA	1.14 (0.38–1.69)
Xinyi Yu	2025	PCS	11,663	49.20%	67.00	TyG index	11.1 (yr)	1.3 (1.03–1.67)	NA
Zhe Wang	2022	PCS	384	61.50%	58.10	HOMA-IR	1.2 (yr)	NA	1.26 (1.1–1.46)
Jianliang Liu	2025	RCS	293	44.70%	60.00	TyG index	1.4–3 (yr)	NA	1.47 (1.01–2.14)
Johnson	2018	PCS	772	43.00%	64.50	HOMA-IR	10.4 (yr)	1.55 (1.35–1.82)	NA
Muhammad	2023	PCS	32,917	50.00%	58.00	TyG index	20 (yr)	1.16 (0.89–1.04)	NA
Jung-Chi Hsu	2023	RCS	28,618	46.90%	64.50	HOMA-IR	4 (yr)	1.24(1.11–1.39)	NA

TyG index, triglyceride-glucose index; eGDR, estimated glucose disposal rate; HOMA-IR, homeostasis model assessment of insulin resistance; RCS, retrospective cohort study; PCS, prospective cohort study.

### 3.3 Risk of bias assessment results for included studies

The ROBINS-I (Risk Of Bias In Non-randomized Studies of Interventions) tool was used to assess the risk of bias in all 30 included cohort studies. Most studies were rated as having moderate risk in the “confounding bias” domain, while the other domains, such as participant selection, intervention classification, and outcome measurement, were mostly rated as low risk. Overall, 26 studies were judged as having a moderate overall risk, and 4 studies were judged as low risk. None of the included studies had a serious risk of bias.

**Figure 2:**
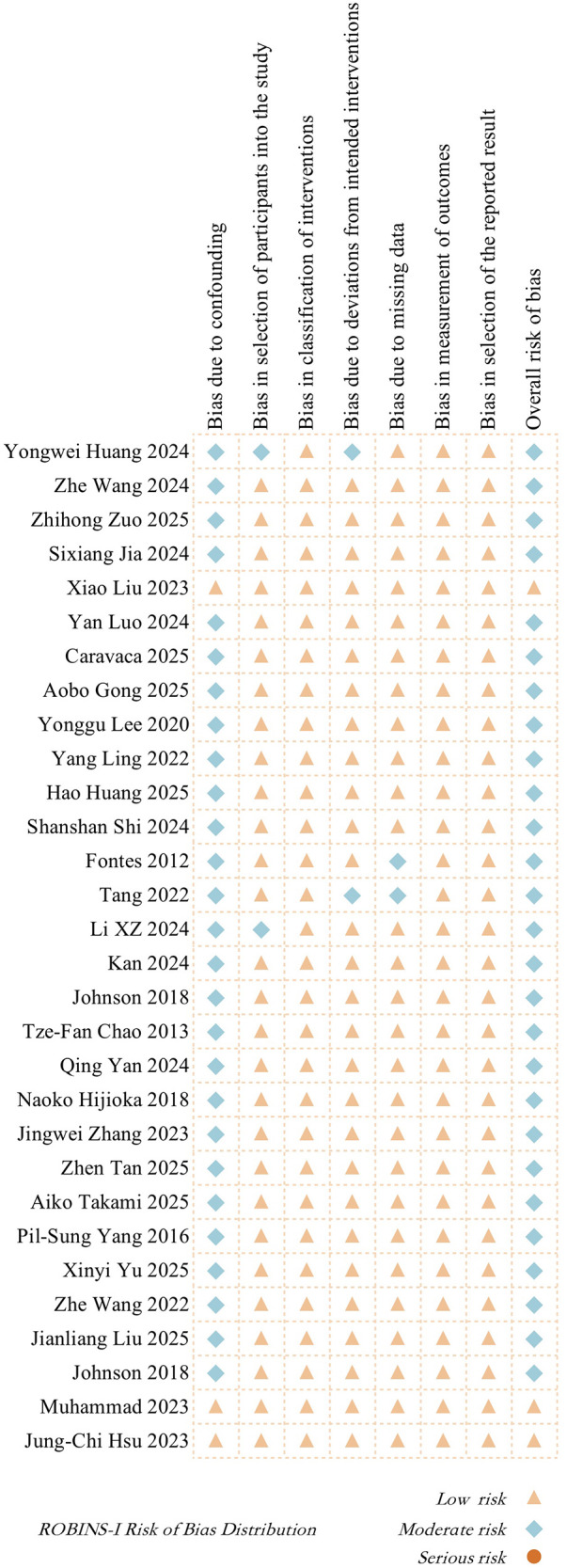
ROBINS-I risk of bias distribution in included studies.

The specific evaluation process is shown in Supplementary Table 3. The risk of bias distribution across different domains for each study is further illustrated in Figure 2, which visually reflects the overall quality status.

### 3.4 Meta-analysis results of insulin resistance and major outcomes of atrial fibrillation

**Figure 3:**
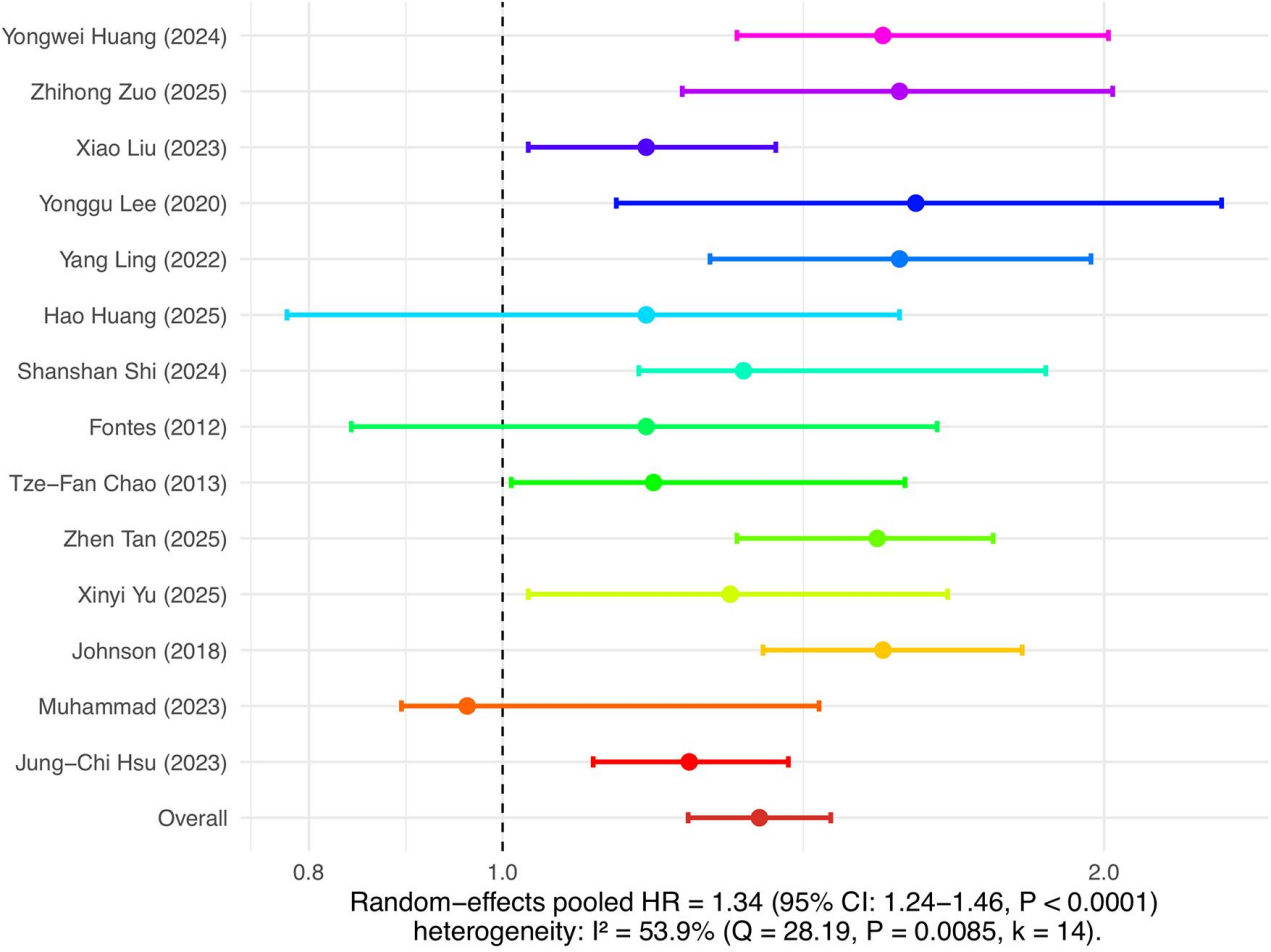
Insulin resistance and risk of atrial fibrillation occurrence.

**Figure 4:**
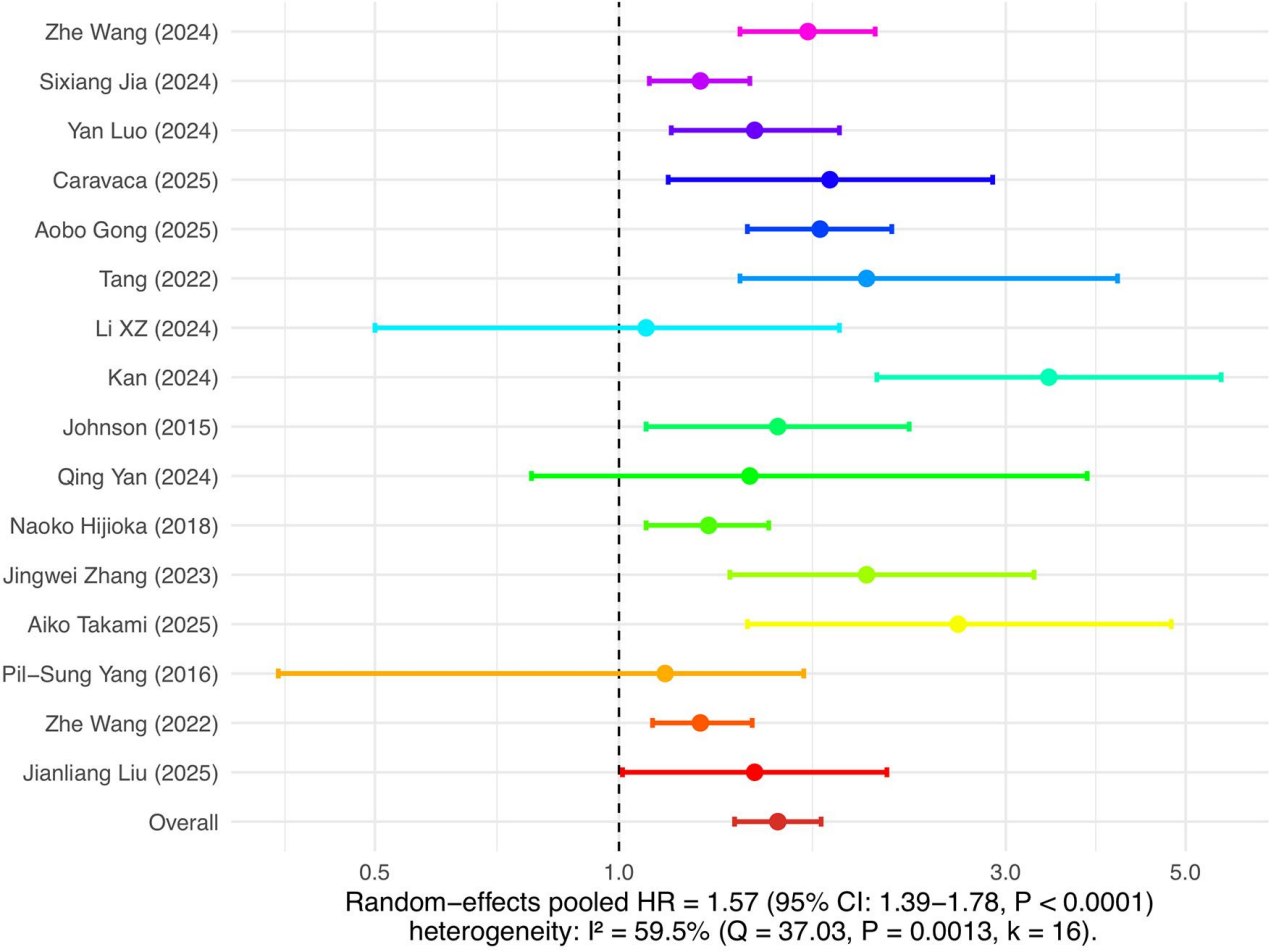
Insulin resistance and risk of atrial fibrillation recurrence.

This study separately analyzed the association between insulin resistance and the risk of new-onset atrial fibrillation as well as the risk of recurrence after ablation. In the analysis of atrial fibrillation incidence risk, 14 cohort studies were included. The combined results of the random effects model showed that insulin resistance was significantly associated with the risk of new-onset atrial fibrillation, with a pooled HR of 1.34 (95% CI: 1.24–1.46, *P* < 0.0001), and heterogeneity I² was 53.9%, indicating moderate heterogeneity among studies (see Figure 3). In the analysis of atrial fibrillation recurrence risk, 16 cohort studies were included, with a pooled HR of 1.57 (95% CI: 1.39–1.78, *P* < 0.0001), and heterogeneity I² was 59.5%, also indicating relatively high heterogeneity (see Figure 4). Among these studies, there was substantial variation in ablation modality (most focused on radiofrequency ablation, with few reporting on cryoballoon or other types), definitions of recurrence (e.g., any episode of atrial arrhythmia >30s, symptomatic AF, or need for repeat ablation), and follow-up duration. Due to incomplete and inconsistent reporting of these variables, further subgroup or sensitivity analyses could not be systematically performed. These results indicate that insulin resistance is not only an important risk factor for the occurrence of atrial fibrillation, but also significantly increases the risk of recurrence after atrial fibrillation ablation.

### 3.5 Publication bias analysis

**Figure 5:**
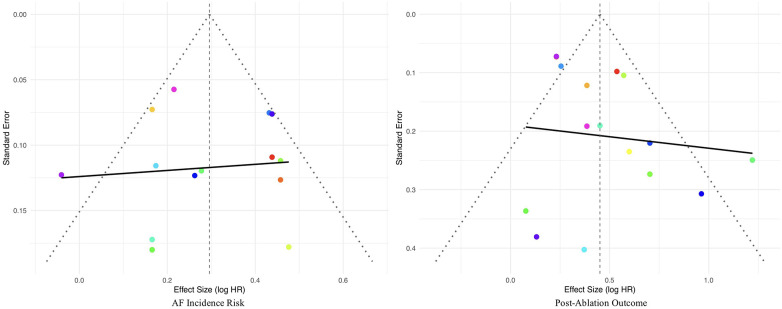
Funnel plots for publication bias.

To assess the potential presence of publication bias, combined funnel plots were constructed for the studies included in the analyses of atrial fibrillation incidence risk and recurrence risk (Figure 5). In the analysis of atrial fibrillation incidence risk, the funnel plot was relatively symmetrical, and neither the Egger's regression test (z = −0.2422, *P* = 0.8086) nor the Begg's test (Kendall's tau = −0.0989, *P* = 0.6672) detected significant publication bias, indicating the pooled effect size was robust. In the analysis of atrial fibrillation recurrence risk, the funnel plot was also generally symmetrical, with the Egger's regression test (z = 1.9581, *P* = 0.0502) approaching but not reaching statistical significance, and the Begg's test (Kendall's tau = 0.1833, *P* = 0.3502) also not statistically significant. The results indicate that no definite publication bias was found in the meta-analyses of the two main outcomes.

#### 3.6.1 Subgroup analysis of the risk of atrial fibrillation occurrence

To explore potential sources of heterogeneity, this study conducted multidimensional subgroup analyses on the association between insulin resistance and the risk of atrial fibrillation occurrence. In the subgroup analysis by study type, both retrospective cohort studies (RCS) and prospective cohort studies (PCS) observed that insulin resistance significantly increased the risk of atrial fibrillation occurrence, but the heterogeneity in both groups remained at a moderate to high level, suggesting that study type had limited explanatory power for heterogeneity and that the association was relatively robust. Stratification by sample size showed that the heterogeneity in the small and large sample size groups was significantly lower than that in the medium sample size group, indicating that the distribution of sample size contributed to the overall heterogeneity. Studies with smaller sample sizes reported a more pronounced and consistent risk increase, and the large sample group also maintained a significant association. In the IR assessment index subgroup, studies using the TyG index had relatively higher heterogeneity (I² = 62.1%), while those using other IR indices (such as HOMA-IR, eGDR) had lower heterogeneity (I² = 49.1%), indicating that although the effect sizes were consistent across different indices, the heterogeneity distribution differed, suggesting that the IR assessment method was an explanatory factor for heterogeneity. Regional stratification analysis found that studies conducted in China had lower heterogeneity and were more consistent compared to those from other countries, indicating that regional factors were also an important source of heterogeneity. In most subgroups, the positive association between insulin resistance and the risk of atrial fibrillation occurrence was consistent and robust, further supporting the main analysis conclusions. Detailed subgroup analysis data are shown in Table 2.

**Table 2:** Subgroup analysis of AF incidence.

Subgroup	Study(k)	HR	I²	Q	*P*	tau²
Study design						
RCS	5	1.39 (1.23–1.58)	52.90%	8.5	0.075	0.0106
PCS	9	1.31 (1.17–1.47)	59.30%	19.67	0.0116	0.0165
Sample size (*n*)						
*n* < 5,000	5	1.53 (1.39–1.68)	0.00%	2.46	0.6512	<0.0001
5,000 < *n* < 10,000	6	1.28 (1.12–1.46)	66.10%	14.74	0.0115	0.0176
*n* > 1,00,000	3	1.24 (1.07–1.44)	0.00%	0.48	0.7886	0
Mean age						
Age>60	6	1.44 (1.30–1.60)	47.70%	9.56	0.0886	0.0076
Age<60	8	1.26 (1.12–1.42)	54.30%	15.31	0.0323	0.0146
Follow up(y)						
y ≥ 11	7	1.28 (1.12–1.46)	59.10%	14.66	0.0231	0.0166
y ≤ 11	7	1.40 (1.26–1.56)	50.00%	12	0.062	0.0089
IR Index						
TyG IR	7	1.33 (1.16–1.52)	62.10%	15.85	0.0146	0.0198
Other IR	7	1.36 (1.22–1.52)	49.10%	11.78	0.067	0.0093
Country						
China	6	1.38 (1.23–1.53)	41.70%	8.57	0.1274	0.0074
Other	8	1.31 (1.15–1.49)	64.30%	19.62	0.0065	0.0201

#### 3.6.2 Subgroup analysis of the risk of atrial fibrillation recurrence

To further explore the sources of heterogeneity in the meta-analysis of atrial fibrillation recurrence risk, subgroup analyses were conducted according to study design, country/region, sample size, IR assessment indices, and follow-up duration. The results showed that country/region, IR assessment indices, and follow-up duration all had significant impacts on both effect size and heterogeneity.

Regional stratification showed that studies from China had a pooled HR of 1.59 (95% CI: 1.36–1.85) with heterogeneity of 66.8%, while studies from other countries had a pooled HR of 1.54 (95% CI: 1.22–1.94) with significantly lower heterogeneity (I² = 41.9%). This suggests greater internal variability among Chinese studies, possibly related to differences in sample composition, diagnostic and treatment processes, IR and AF management strategies, baseline disease spectrum, and other factors. Although there were fewer international studies, their effect sizes were basically consistent with those in China, and the results were more consistent, indicating that ethnic and regional specificity is worthy of attention.

Subgroup analysis of IR assessment indices showed that studies using the TyG index had a pooled HR of 1.69 (95% CI: 1.45–1.97) with relatively high heterogeneity (I² = 62.0%), while those using other IR indices (such as HOMA-IR, etc.) had a pooled HR of 1.31 (95% CI: 1.18–1.45) with markedly reduced heterogeneity (I² = 26.2%). This indicates that the choice of IR index has an important impact on the stability and interpretability of meta-analysis results. The TyG index, as an emerging and easily applicable index closely related to cardiovascular outcomes, demonstrates a stronger risk prediction capability, but there is also greater variability in research methods, cut-off values, and patient composition. Traditional indices (such as HOMA-IR) show better consistency of results and lower heterogeneity.

Stratified analysis by follow-up duration found that studies with follow-up ≥2 years had a pooled HR of 1.62 (95% CI: 1.38–1.91) with heterogeneity of 49.4%; those with follow-up <2 years had a pooled HR of 1.53 (95% CI: 1.22–1.92) with significantly increased heterogeneity (I² = 70.7%). This suggests that studies with shorter follow-up durations have more variable results, possibly related to cumulative events, definitions of AF recurrence, and case loss rates, while longer follow-up provides a more stable effect size and better represents the true risk.

Country/region, IR assessment methods, and follow-up duration can all effectively explain the heterogeneity observed in the analysis of AF recurrence risk. Future study designs should pay more attention to stratification and standardization of these factors to improve comparability and the clinical significance of conclusions. Detailed subgroup analysis results are shown in Table 3.

**Table 3:** Subgroup analysis of AF recurrence.

Subgroup	Study(k)	HR	I²	Q	*P*	tau²
Study design						
PCS	4	1.29 (1.16–1.44)	0.00%	2.35	0.5024	0
RCS	12	1.67 (1.44–1.94)	59.77%	27.3	0.0041	0.032
Country						
China	11	1.59 (1.36–1.85)	66.77%	30.04	0.0008	0.0378
Other	5	1.54 (1.22–1.94)	41.88%	6.87	0.1429	0.027
Sample size (*n*)						
*n* < 500	8	1.62 (1.27–2.06)	65.88%	20.44	0.0047	0.0746
*n* > 500	8	1.56 (1.36–1.79)	53.44%	15.03	0.0356	0.0164
IR index						
TyG IR	10	1.69 (1.45–1.97)	62.00%	23.69	0.0048	0.0318
Other IR	6	1.31 (1.18–1.45)	26.22%	6.78	0.2377	0.0001
Follow up(y)						
y ≥ 2	9	1.62 (1.38–1.91)	49.44%	15.81	0.0452	0.0228
y < 2	7	1.53 (1.22–1.92)	70.66%	20.42	0.0023	0.0615

### 3.7 Sensitivity analysis

**Figure 6:**
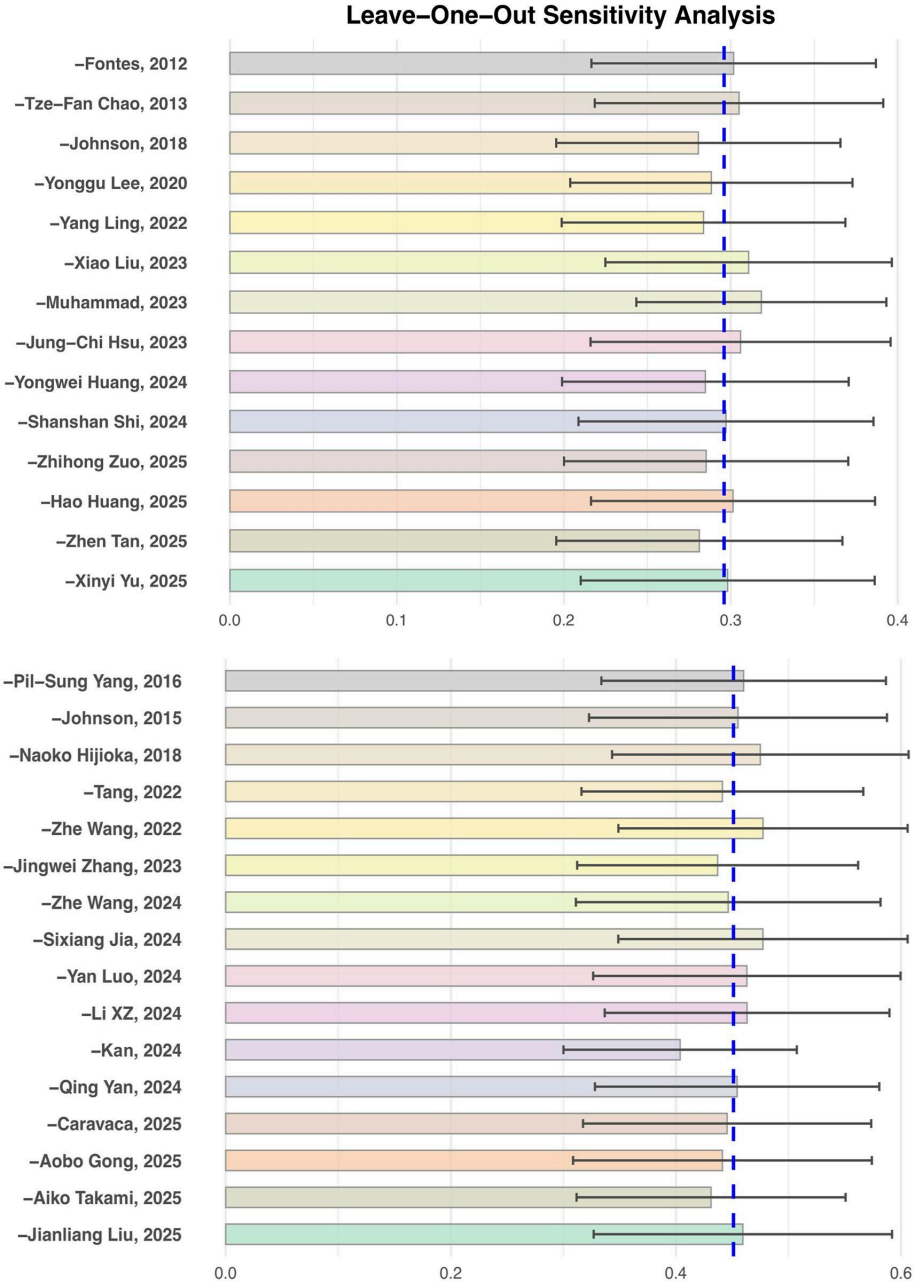
Leave-One-Out sensitivity analysis of insulin resistance and the risk of atrial fibrillation occurrence and recurrence.

To test the robustness of the main outcome analysis results, leave-one-out sensitivity analyses were performed for both the risk of atrial fibrillation occurrence and the risk of recurrence (see Figure 6). The results showed that no matter which individual study was excluded, the pooled effect size (HR) did not change substantially, and the main analysis results for both outcomes were not significantly influenced by any single study. This indicates that the results of this meta-analysis are robust and the analytical conclusions are reliable.

### 3.8 Meta-regression analysis

To further explore potential sources of heterogeneity, this study used follow-up duration and mean age as covariates to perform meta-regression analyses for both the risk of atrial fibrillation occurrence and recurrence.

In the meta-regression for the risk of atrial fibrillation occurrence, follow-up duration had a statistically significant impact on the pooled effect value (regression coefficient = −0.0111, *P* = 0.0481), with an R² of 20.71%, suggesting that with each additional year of follow-up, the association between insulin resistance and the risk of atrial fibrillation occurrence tends to gradually weaken. This result has important clinical and methodological implications: on one hand, it indicates that the “short-term” risk impact of IR on atrial fibrillation may be more pronounced, while some risk effects may be diluted or influenced by other factors over longer follow-up; on the other hand, it suggests that future related studies should pay attention to controlling for and reporting the impact of follow-up duration to achieve more consistent effect estimates. When mean age was used as a covariate, the meta-regression showed a positive trend (regression coefficient = 0.0114), but it did not reach statistical significance (*P* = 0.1331), with an R² of 19.36%. This suggests that within the range of included study samples, mean age was not a major source of heterogeneity in the relationship between IR and new-onset atrial fibrillation risk. It indicates that the positive association between IR and atrial fibrillation is relatively consistent across different age groups, with no evident age-modification effect observed.

**Figure 7:**
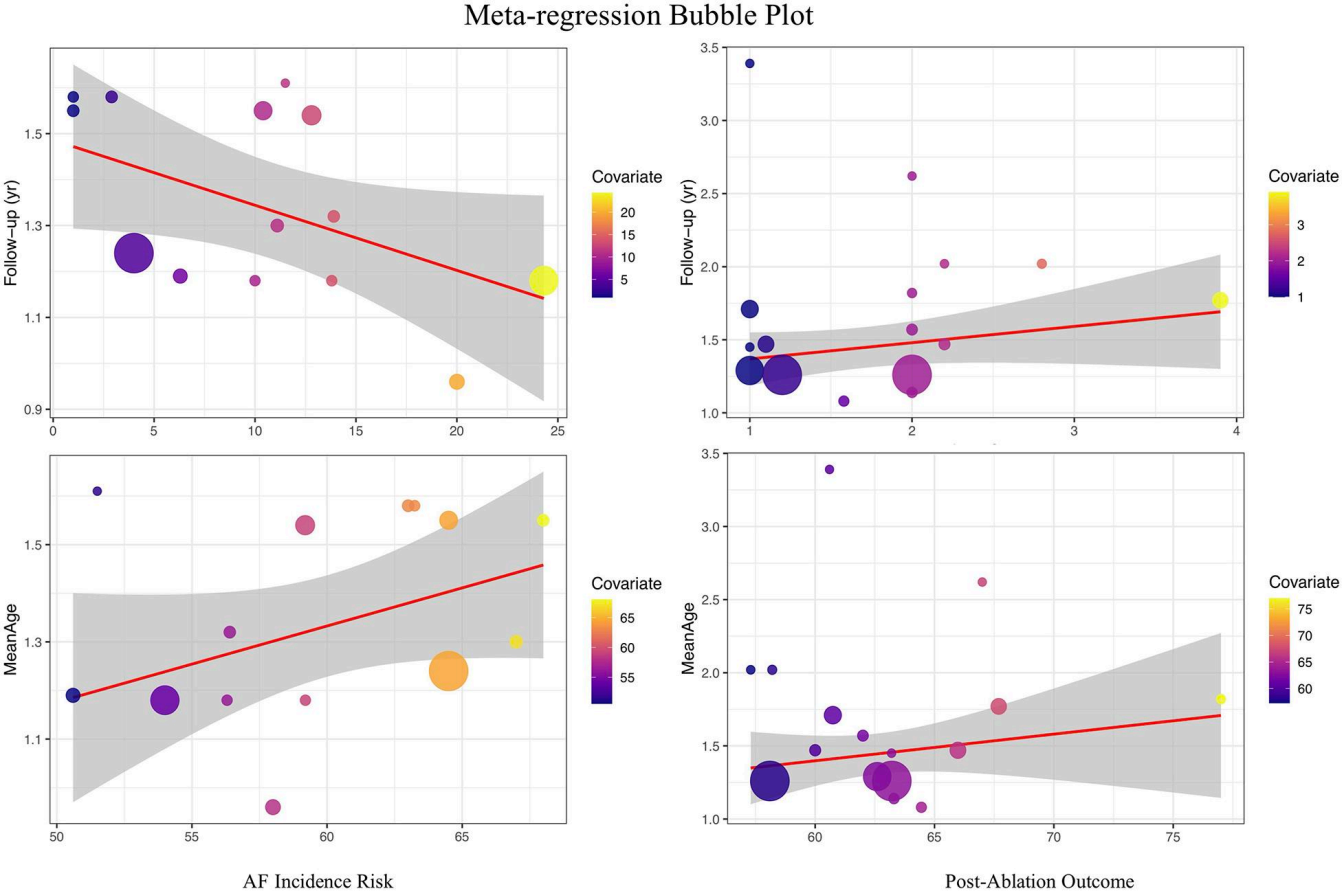
Meta-Regression plot of the association between insulin resistance and atrial fibrillation risk.

In the meta-regression analysis for the risk of atrial fibrillation recurrence, neither follow-up duration (regression coefficient = 0.0565, *P* = 0.4413, R² = 0%) nor mean age (regression coefficient = 0.0051, *P* = 0.7285, R² = 0%) showed significant explanatory power for the pooled effect value or heterogeneity. This indicates that the effect size for recurrence risk remains highly consistent across studies with different ages and follow-up durations, suggesting that the impact of IR on the risk of atrial fibrillation recurrence has broad applicability and that this association is not easily affected by differences in study population age structure or follow-up duration (see Figure 7).

Unfortunately, further meta-regression analyses for other clinically important variables—such as diabetes status, body mass index (BMI), presence of comorbidities, ablation type, and ethnicity—were not feasible due to insufficient and inconsistent reporting of these factors across the included studies. This data limitation has constrained our ability to comprehensively explore all potential sources of heterogeneity. Future research with standardized and detailed reporting of key clinical characteristics will be essential for enabling more in-depth investigation into the determinants of heterogeneity in this field.

### 3.9 GRADE quality of evidence

All included studies were cohort designs, with an initial evidence level rated as low. Considering risk of bias, consistency, indirectness, precision, and publication bias, the quality of evidence for both main outcomes was assessed as “moderate.” The main reasons for downgrading were moderate risk of bias and the presence of certain heterogeneity among studies. Detailed assessment information is provided in Supplementary Table 4.

## 4 Discussion

### 4.1 Summary of main findings

A total of 30 cohort analyses were included in this study, covering multiple countries and regions including China, the United States, and Europe, with a total sample size exceeding 800,000 cases. The results showed that insulin resistance significantly increased the risk of new-onset atrial fibrillation (pooled HR = 1.34, 95% CI: 1.24–1.46), and was also an important independent risk factor for recurrence after atrial fibrillation ablation (pooled HR = 1.57, 95% CI: 1.39–1.78). These conclusions demonstrated good robustness in multidimensional subgroup analyses and sensitivity analyses. This study systematically reviewed the association between IR and the “dual outcomes” of atrial fibrillation, providing new evidence-based support for clinical risk stratification and management strategies.

We also performed meta-regression analyses for age and follow-up duration as described above; however, analysis for other variables was not feasible due to incomplete reporting.

### 4.2 Mechanistic basis of insulin resistance promoting new-onset and recurrent atrial fibrillation after ablation

Multiple studies have shown that inflammatory response and oxidative stress are the core mechanisms by which insulin resistance (IR) promotes new-onset atrial fibrillation and recurrence after ablation. In the IR state, adipocyte inflammation is enhanced, free fatty acids remain persistently elevated, and multiple inflammatory factors (such as TNF-α, IL-6) are activated, which promote the activation of atrial fibroblasts, induce atrial structural remodeling and fibrosis, and aggravate cardiac tissue injury and remodeling (7–10). Multiple studies have shown that inflammatory response, oxidative stress, and atrial structural remodeling are the main pathways linking insulin resistance (IR) to both new-onset atrial fibrillation and recurrence after ablation. In the IR state, enhanced adipocyte inflammation and activation of cytokines such as TNF-α and IL-6 can promote atrial fibrosis and tissue remodeling, increasing susceptibility to arrhythmia (7–10). Additionally, IR-induced oxidative stress may contribute to myocyte apoptosis and electrical instability, further facilitating the development and recurrence of atrial fibrillation (7–13). Clinical and experimental studies support these mechanisms and indicate that metabolic abnormalities associated with IR can jointly increase the risk of adverse atrial remodeling and arrhythmogenicity (9–11, 14–26).

In terms of electrophysiology, IR and metabolic abnormalities can lead to delayed atrial conduction and increased heterogeneity, specifically manifested as increased P-wave dispersion, prolonged maximum P-wave duration, and prolonged left atrial electromechanical interval (PA-PDI), all of which are clinical indicators reflecting atrial electrophysiological remodeling and susceptibility to atrial fibrillation (12, 13). Relevant studies have found that PA-PDI is positively correlated with IR levels, and both P-wave dispersion and Pmax are independently associated with HOMA-IR, suggesting that IR directly affects atrial conduction and electrical stability, thereby increasing the risk of new-onset and recurrent atrial fibrillation after ablation (11–13, 23, 27–36). Basic research has also revealed that IR status can cause abnormal expression of connexin proteins and remodeling of sodium, potassium, and calcium channels, weakening atrial conduction velocity and synchrony at the molecular level and promoting the occurrence of atrial reentry and ectopic excitation (10, 11).

Clinical studies have found that IR often coexists with components of metabolic syndrome such as obesity, hypertension, and dyslipidemia, and that the synergistic effect of multiple cardiovascular risk factors further increases the risk of new-onset and recurrent atrial fibrillation. Epidemiological studies have confirmed that the greater the number of metabolic syndrome components, the higher the risk of atrial fibrillation occurrence and postoperative recurrence, with IR being the core mechanism driving the aggregation of these risk factors (9, 10, 18, 28–39). Some studies have also found that postoperative patients with IR have persistently elevated inflammatory markers such as high-sensitivity CRP, suggesting that this is not conducive to the long-term recovery of atrial electrical stability (7, 9).

**Figure 8:**
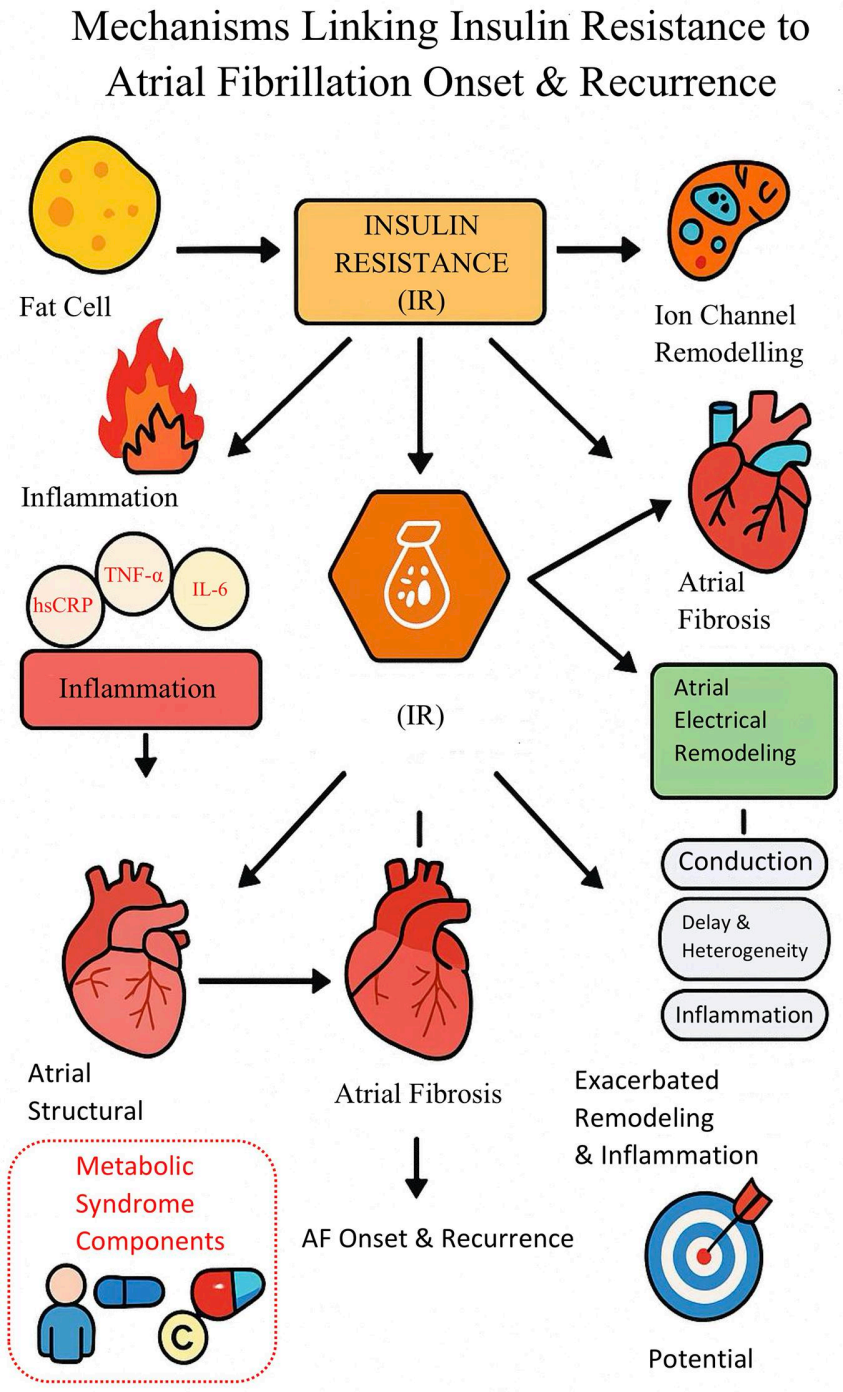
Mechanistic pathway diagram of insulin resistance promoting New-onset and recurrent atrial fibrillation.

Current evidence suggests that IR, through multiple mechanisms including inflammation, structural remodeling, and electrophysiological remodeling, may play a role throughout the entire process of new-onset and recurrent atrial fibrillation after ablation, making it a potential management target worthy of attention (7–13) (Figure 8).

#### 4.3.1 Insulin resistance may become an important target for atrial fibrillation risk management

Insulin resistance (IR) not only plays a deep role in the occurrence and recurrence of atrial fibrillation after ablation, but also runs through the entire course of atrial fibrillation via multiple pathways such as inflammation, structural remodeling, and electrophysiological abnormalities. Our study found that the impact of IR on atrial fibrillation risk remained robust across multiple subgroups and sensitivity analyses, suggesting that it is not only an independent risk factor but may also represent a “blind spot” in traditional risk assessment systems. Current clinical practice often focuses on clear cardiovascular risk factors such as diabetes, making it difficult to identify individuals at high potential risk who have normal blood glucose but exhibit IR. In fact, IR often appears earlier than the clinical diagnosis of diabetes and, in the early stages of disease, already promotes atrial matrix injury and dysfunction, laying the groundwork for the occurrence and recurrence of atrial fibrillation (8, 10).

Incorporating IR into atrial fibrillation risk management helps to address the shortcomings of existing prevention and control systems, enabling earlier and more precise stratification of risk. Populations with IR-related conditions such as obesity, metabolic syndrome, and hypertension are “hard-hit” groups for the incidence and recurrence of atrial fibrillation and are often missed due to inadequate traditional screening (9, 37). Lin et al. found that even in the absence of diabetes, elevated IR levels result in a continuous accumulation of atrial fibrillation risk (10). Giraldo-Gonzalez et al. and Awashra et al. further pointed out that dynamic monitoring and intervention of IR help to identify high-risk patients early in the community, primary care, and chronic disease management systems, optimizing follow-up and resource allocation (8, 9).

Therefore, it is recommended that IR indicators such as HOMA-IR and TyG index be incorporated into the routine assessment process in both primary and secondary prevention systems for atrial fibrillation, advancing IR management, and providing more forward-looking warnings and individualized prevention and control measures for high-risk populations (8–10, 37, 40, 41).

#### 4.3.2 Promoting the “metabolic” transformation of atrial fibrillation management processes

Although the current atrial fibrillation (AF) management system is continuously improving, traditional strategies still focus mainly on electrophysiological monitoring, anticoagulation prevention, and rhythm control, while metabolic abnormalities are far from adequately emphasized in clinical practice. This study and prior evidence suggest that insulin resistance and its associated metabolic disorders may not only be related to the initial onset of atrial fibrillation, but could also contribute to the risk of recurrence after ablation. These findings raise the possibility that strategies focused exclusively on electrocardiographic and anticoagulation measures may not fully address the complex mechanisms underlying AF. The presence of IR and metabolic abnormalities may serve as “invisible drivers” of atrial structural and electrophysiological remodeling, potentially influencing the overall effectiveness of comprehensive AF management (7, 8, 10, 11).

In recent years, multiple studies have proposed that dynamic assessment of metabolic abnormalities, especially IR, could be considered as a supplement to the current management of atrial fibrillation, potentially contributing to a more integrated “metabolism–electrophysiology” model (8, 10, 37). Bell & Goncalves et al. observed that, in populations with metabolic abnormalities such as obesity and metabolic syndrome, traditional AF treatments alone may be associated with higher long-term recurrence rates, whereas comprehensive management that includes metabolic factors such as IR may be linked to improved outcomes (37). Giraldo-Gonzalez et al. further suggested that a “cardiac–metabolic–inflammatory” collaborative assessment could offer a theoretical and practical basis for more refined risk stratification and optimized intervention pathways for atrial fibrillation (8).

In the future, comprehensive management of atrial fibrillation may benefit from the integration of IR and metabolic abnormality assessment into routine care, potentially helping to overcome some of the limitations of traditional “electrophysiology-oriented” approaches. However, as current evidence is largely observational, further prospective and interventional studies are needed to confirm whether such strategies can lead to sustained improvements in prevention, efficacy, and individualized management of atrial fibrillation (7, 8, 10, 11, 37).

#### 4.3.3 Personalized precision management and the construction of novel risk tools

Insulin resistance (IR), as a risk factor for both new-onset and recurrent atrial fibrillation after ablation, can be used not only for risk stratification but also provides a solid evidence-based foundation for precise intervention and intelligent management of atrial fibrillation in the future. Based on this study and related literature, it is recommended that IR levels be incorporated into the entire management system for AF patients, promoting the organic integration of stratified warning, precise intervention, and dynamic risk prediction.

In clinical practice, patients can be stratified according to IR levels, and comprehensive intervention strategies can be customized focusing on lifestyle optimization (diet structure, exercise prescription, scientific weight loss), pharmacological intervention, and comorbidity management, to maximize the reduction of atrial inflammation, structural remodeling, and electrophysiological abnormalities (8–10, 37). Especially for patients undergoing AF ablation, dynamic monitoring of IR indicators before and after the procedure not only helps to optimize surgical timing and indications, but also provides real-time reference for perioperative management and postoperative recurrence risk control (7, 8). For patients whose IR improves or worsens over time, differentiated follow-up frequencies and individualized management plans should be developed to enhance long-term outcomes and quality of life.

In future AF prevention and control practices, it is recommended to develop and promote multi-parameter risk prediction models and clinical decision-making tools that integrate IR indicators. By introducing technologies such as big data and artificial intelligence, IR can be analyzed together with other cardiovascular risk factors, imaging, and biomarker data to achieve dynamic and intelligent management of AF onset, recurrence, and long-term prognosis (8–10, 37, 40–42). This model is expected to improve the early identification and stratification efficiency of high-risk populations, and will also promote the deep implementation of precision medicine in the full-cycle management of AF, providing theoretical basis and practical reference for the development of new AF prevention and control guidelines and follow-up procedures centered on IR in the future. The promotion and application of these strategies still require further optimization and validation based on different population characteristics and prospective clinical studies.

### 4.4 Contributions of studies from mainland China and Asian populations and the value of regionalized management

#### 4.4.1 Progress of studies from mainland China and the impact of population characteristics on results

Recent studies have shown that, with the increasingly prominent trends of chronic disease and atrial fibrillation prevalence, research from mainland China on the relationship between insulin resistance (IR) and related metabolic indicators (such as the TyG index) and the risk of atrial fibrillation has significantly increased. Multiple large-sample, regional cohort studies have provided a solid data foundation for this meta-analysis, making the results more representative (40–42).

The prevalence of metabolic abnormalities (such as diabetes and metabolic syndrome) is high in the Chinese population, and the role of IR in the pathogenesis of atrial fibrillation is even more prominent. Domestic studies have confirmed that non-insulin-based indicators such as TyG and METS-IR are not only closely related to the occurrence of atrial fibrillation, but can also predict recurrence after ablation and adverse cardiovascular outcomes (23–29, 40, 42). Gong et al. showed that the TyG index is independently associated with major adverse cardiovascular events in patients with atrial fibrillation and can optimize the risk prediction of the CHA₂DS₂-VASc scoring model (41).

This study included a large amount of data from mainland China, making the results more consistent with the actual characteristics of the Chinese population, and also reflecting the particularities of diet, genetics, and disease spectrum in mainland China. The advantage of dynamically monitoring the cumulative TyG index for predicting the risk of atrial fibrillation recurrence in China has been confirmed (42). Currently, the management of atrial fibrillation and IR interventions in China still faces challenges such as insufficient early screening and low recognition rates of metabolic abnormalities. However, the continuous accumulation of high-quality studies provides an evidence base for formulating China-specific risk prediction and intervention strategies. In the future, prospective studies should be strengthened to enhance the influence of Chinese evidence in international guidelines.

#### 4.4.2 Regionalized atrial fibrillation management strategies and the clinical value of IR indicators

The results of this study provide a basis for formulating more “regionalized” atrial fibrillation management strategies for Chinese and Asian populations. Compared with Western countries, Chinese and Asian populations have a higher prevalence of metabolic abnormalities (such as diabetes, metabolic syndrome, and insulin resistance), and patients with atrial fibrillation often have multiple concomitant metabolic disorders, with significant differences in disease spectrum and risk factor composition (40–42). Against this background, IR indicators (such as the TyG index and METS-IR) are not only easy to obtain, economical, and practical, but also demonstrate higher clinical application value in atrial fibrillation risk prediction, stratified management, and individualized prevention and treatment (41, 42).

With the continuous enrichment of relevant evidence-based research, an increasing amount of clinical practice evidence from China and Asia supports the incorporation of IR-related indicators into atrial fibrillation risk assessment and management processes. This is of great significance for optimizing high-risk patient screening, developing combined metabolic intervention measures, and promoting the integration of local atrial fibrillation management guidelines with international standards. In the future, it is necessary to further explore the pathways for promoting the use of IR indicators in the comprehensive management of atrial fibrillation, taking into account the characteristics of different regions and different patient subtypes, in order to continuously improve the level of atrial fibrillation prevention and treatment in China and enhance its international influence.

It should be emphasized that although growing evidence from China and other Asian countries highlights the promising value of IR-related indices in risk assessment and management, their routine clinical adoption still requires further validation in prospective studies and endorsement by international guidelines.

#### 4.4.3 Clinical implications and guideline context

In current clinical practice, management of atrial fibrillation is primarily guided by established cardiovascular risk factors, and IR-related indices—such as the TyG index and HOMA-IR—are not yet incorporated into standard clinical guidelines. However, accumulating evidence, including the results of our meta-analysis, suggests that these markers may facilitate early identification and risk stratification of patients with elevated metabolic and arrhythmic risk, particularly in populations with a high prevalence of metabolic disorders.

While IR-related indices represent practical and accessible supplementary tools for individualized risk assessment—especially in patients without overt diabetes but with metabolic syndrome features—the observational nature of most supporting studies and the moderate certainty of our findings (as rated by GRADE) mean that current evidence does not support their routine clinical use. Further prospective studies and broader consensus in guideline development will be necessary before these indices can be formally incorporated into standardized atrial fibrillation management, both regionally and globally.

Beyond IR-related indices, recent international studies have explored additional metabolic and echocardiographic markers for predicting and managing atrial fibrillation risk, such as the leukoglycemic index, FIB-4, systemic immune-inflammation index, and transmitral A-wave acceleration time (43, 44). Although these emerging markers require further validation, they reflect a growing trend toward multi-parameter risk stratification and may ultimately refine individualized management strategies for diverse patient populations.

### 4.5 Limitations and prospects of the results

Although this study included a large number of high-quality studies from both domestic and international sources, there are still some unavoidable limitations, and the interpretation of the relevant results should remain cautious.

First, there is a certain degree of heterogeneity among the included studies, and some of the heterogeneity has not been fully explained. Although potential influencing factors such as study design type, sample size, follow-up duration, IR assessment indicators, and regions were explored through subgroup analysis and meta-regression, and heterogeneity was partially reduced within some subgroups, the overall heterogeneity remained relatively high. This indicates that there may still be unidentified confounding factors or methodological differences among studies that have not been fully captured. In particular, differences in IR assessment methods, baseline patient characteristics, intervention strategies, and follow-up periods may have influenced the pooled effect estimates. Additionally, for studies on recurrence after ablation, heterogeneity may also be attributed to differences in ablation modality (e.g., radiofrequency vs. cryoballoon), definitions of recurrence, and follow-up protocols across studies. The lack of detailed and consistent reporting on these ablation-related characteristics limited our ability to perform further subgroup or sensitivity analyses, and this should be considered when interpreting the pooled estimates and their applicability to clinical practice. Although the results demonstrated a certain degree of robustness across multiple sensitivity and subgroup analyses, they should still be interpreted with caution to avoid overgeneralization.

Second, important limitations related to study design and causal inference should be acknowledged. All included studies in this meta-analysis were observational in nature, primarily cohort studies. Despite comprehensive multivariable adjustment and rigorous risk-of-bias evaluation, the inherent limitations of observational research—including the possibility of residual confounding and selection bias—cannot be entirely eliminated. As such, the observed associations between insulin resistance and both new-onset atrial fibrillation and post-ablation recurrence should be interpreted as correlational rather than causal. This limitation is further reflected in our GRADE assessment, where the overall quality of evidence for the primary outcomes was rated as moderate. Future large-scale, prospective, and interventional studies will be essential to establish causality and to confirm whether modification of insulin resistance can directly improve atrial fibrillation outcomes.

Third, data- and methodology-related limitations should be considered. Some included studies had relatively small sample sizes or were conducted at single centers, potentially limiting statistical power and generalizability. Differences in confounder adjustment strategies, data completeness, outcome definitions, and follow-up durations across studies may have contributed to underestimation or overestimation of effect sizes.

Fourth, this meta-analysis included studies that used a variety of insulin resistance (IR) indices—such as the triglyceride-glucose (TyG) index, HOMA-IR, and eGDR—with differences in calculation methods, physiological underpinnings, and clinical applicability. The choice of IR indicator was often influenced by laboratory resources, study design, and population characteristics. In addition, there was substantial heterogeneity in the cut-off values used to define IR positivity across studies, with thresholds determined by local population data or statistical criteria rather than standardized international references. Such methodological variability may affect both the identification of high-risk individuals and the comparability of effect estimates between studies. Moreover, the predictive value of each IR index may vary by ethnicity and baseline metabolic status; for example, TyG may perform better in East Asian populations, whereas HOMA-IR is more commonly validated in Western cohorts. These differences should be considered when interpreting our findings and may have contributed to the observed heterogeneity. Future research should prioritize the development of standardized, widely applicable definitions and direct head-to-head comparisons of different IR indices in diverse populations.

In view of these limitations, future research should further optimize study design, strengthen long-term and standardized follow-up, and promote harmonization in the measurement and reporting of IR-related indicators. Although we performed meta-regression analyses for mean age and follow-up duration, additional exploration of other important sources of heterogeneity—such as diabetes status, BMI, comorbidities, ablation type, and ethnicity—was not feasible due to limited and inconsistent reporting in the included studies. This constraint may have contributed to residual unexplained heterogeneity and should be considered when interpreting our results. Comprehensive and standardized data collection and reporting, as well as high-quality prospective and mechanistic studies, will be essential to provide a more robust theoretical foundation and practical guidance for individualized risk stratification and precision prevention strategies in atrial fibrillation.

## 5 Conclusion

There is a certain association between insulin resistance and the risk of new-onset atrial fibrillation as well as recurrence after ablation. Relevant IR indicators (such as the TyG index, HOMA-IR, etc.) have shown potential value in atrial fibrillation risk assessment, but current research is still limited by considerable heterogeneity and varying evidence quality. In the future, more high-quality studies on IR-related mechanisms, intervention effects, and indicator standardization are needed to provide a stronger evidence-based foundation for precise stratification and individualized management of atrial fibrillation.

## Data Availability

The original contributions presented in the study are included in the article/Supplementary Material, further inquiries can be directed to the corresponding author.
