# Supplementary material for: Insulin resistance and atrial fibrillation: from disease onset to post-ablation outcomes: a systematic review and meta-analysis
Source: Front Cardiovasc Med. 2026 Jan 8;12:1700730. doi: 10.3389/fcvm.2025.1700730 (PMC12823920; doi:10.3389/fcvm.2025.1700730)
Supplement: Supplementary file 1 [file Table1.docx]

| Supplementary Table 1. Search strategy | | |
| --- | --- | --- |
| Database | Search strategy | Results |
| PubMed | ( "Insulin Resistance"[Mesh] OR "insulin resistance" OR "insulin sensitivity" OR "insulin sensitivity index" OR "insulin resistance index" OR "HOMA-IR" OR "homeostasis model assessment" OR "TyG index" OR "triglyceride-glucose index" OR "triglyceride glucose index" OR "QUICKI" OR "quantitative insulin sensitivity check index" OR "Matsuda index" OR "Gutt index" OR "fasting insulin" OR "fasting glucose" OR "insulin suppression test" OR "hyperinsulinemic-euglycemic clamp" OR "surrogate marker" OR "impaired insulin sensitivity" OR "impaired glucose tolerance" OR "metabolic syndrome")AND( "Atrial Fibrillation"[Mesh] OR "atrial fibrillation" OR "AF" OR "nonvalvular atrial fibrillation" OR "paroxysmal atrial fibrillation" OR "persistent atrial fibrillation")AND( "Ablation"[Mesh] OR "catheter ablation" OR "radiofrequency ablation" OR "pulmonary vein isolation" OR "AF ablation" OR "atrial fibrillation ablation" OR "surgical ablation" OR "cryoablation" OR "maze procedure" OR "recurrence" OR "outcome" OR "prognosis" OR "relapse" OR "follow-up") | 413 |
| Embase | ('insulin resistance'/exp OR 'insulin resistance' OR 'insulin sensitivity' OR 'insulin sensitivity index' OR 'insulin resistance index' OR 'homa ir' OR 'homeostasis model assessment' OR 'tyg index' OR 'triglyceride-glucose index' OR 'triglyceride glucose index' OR 'quicki' OR 'quantitative insulin sensitivity check index' OR 'matsuda index' OR 'gutt index' OR 'fasting insulin' OR 'fasting glucose' OR 'insulin suppression test' OR 'hyperinsulinemic euglycemic clamp' OR 'surrogate marker' OR 'impaired insulin sensitivity' OR 'impaired glucose tolerance' OR 'metabolic syndrome'/exp OR 'metabolic syndrome')AND('atrial fibrillation'/exp OR 'atrial fibrillation' OR 'af' OR 'nonvalvular atrial fibrillation' OR 'paroxysmal atrial fibrillation' OR 'persistent atrial fibrillation')AND('catheter ablation'/exp OR 'ablation'/exp OR 'catheter ablation' OR 'radiofrequency ablation' OR 'pulmonary vein isolation' OR 'af ablation' OR 'atrial fibrillation ablation' OR 'surgical ablation' OR 'cryoablation' OR 'maze procedure' OR 'recurrence' OR 'outcome' OR 'prognosis' OR 'relapse' OR 'follow-up') | 1739 |
| Web of Science | ( "insulin resistance" OR "insulin sensitivity" OR "insulin sensitivity index" OR "insulin resistance index" OR "HOMA-IR" OR "homeostasis model assessment" OR "TyG index" OR "triglyceride-glucose index" OR "triglyceride glucose index" OR "QUICKI" OR "quantitative insulin sensitivity check index" OR "Matsuda index" OR "Gutt index" OR "fasting insulin" OR "fasting glucose" OR "insulin suppression test" OR "hyperinsulinemic euglycemic clamp" OR "surrogate marker" OR "impaired insulin sensitivity" OR "impaired glucose tolerance" OR "metabolic syndrome")AND( "atrial fibrillation" OR "AF" OR "nonvalvular atrial fibrillation" OR "paroxysmal atrial fibrillation" OR "persistent atrial fibrillation")AND( "catheter ablation" OR "ablation" OR "radiofrequency ablation" OR "pulmonary vein isolation" OR "AF ablation" OR "atrial fibrillation ablation" OR "surgical ablation" OR "cryoablation" OR "maze procedure" OR "recurrence" OR "outcome" OR "prognosis" OR "relapse" OR "follow-up") | 561 |
| DATE：2025-08-24 | | |
