# Supplementary material for: Insulin resistance and atrial fibrillation: from disease onset to post-ablation outcomes: a systematic review and meta-analysis
Source: Front Cardiovasc Med. 2026 Jan 8;12:1700730. doi: 10.3389/fcvm.2025.1700730 (PMC12823920; doi:10.3389/fcvm.2025.1700730)
Supplement: Supplementary file 2 [file Table2.docx]

| Supplementary Table 4.GRADE evidence profile table | | | | | | | | |
| --- | --- | --- | --- | --- | --- | --- | --- | --- |
| Outcome | Study Design | Risk of Bias | Consistency | Indirectness | Precision | Publication Bias | Overall Quality (GRADE) | Main Reason for Downgrading |
| Incident AF risk | Cohort Study | Moderate | Moderate | None | High | None | Moderate | Risk of bias, heterogeneity |
| AF recurrence after ablation | Cohort Study | Moderate | Moderate | None | High | None | Moderate | Risk of bias, heterogeneity |
