# Supplementary material for: Insulin resistance and atrial fibrillation: from disease onset to post-ablation outcomes: a systematic review and meta-analysis
Source: Front Cardiovasc Med. 2026 Jan 8;12:1700730. doi: 10.3389/fcvm.2025.1700730 (PMC12823920; doi:10.3389/fcvm.2025.1700730)
Supplement: Supplementary file 3 [file Table3.docx]

Supplementary Table 2

| First Author | Year | Country | Study Design | Sample Size | Male % | Mean Age | IR Index | Follow-up (yr) | Ablation Type | Incident AF (n/N) | Recurrence (n/N) | AF Incidence Risk  (HR,95%CI) | Post-Ablation Outcome  (HR,95%CI) |
| --- | --- | --- | --- | --- | --- | --- | --- | --- | --- | --- | --- | --- | --- |
| Yongwei Huang | 2024 | China | RCS | 1,707 | 55.42% | 68.00 | TyG index | 1 (yr) | AF-RFA | NA | 无 | 1.55(1.31 - 2.01) | NA |
| Zhe Wang | 2024 | China | RCS | 2,242 | 63.38% | 60.74 | TyG index | 1 (yr) | AF-RFA | NA | 711 | NA | 1.71(1.41–2.07) |
| Zhihong Zuo | 2025 | China | RCS | 4,276 | 59.71% | 63.24 | TyG index | 1 (yr) | NA | 764 | NA | 1.58(1.23 - 2.02) | NA |
| Sixiang Jia | 2024 | China | RCS | 997 | 63.19% | 63.21 | TyG index | 1-3 (yr) | AF-RFA | NA | 200 | NA | 1.26(1.09–1.45) |
| Xiao Liu | 2023 | USA | PCS | 11,851 | 44.40% | 54.00 | TyG index | 24.3 (yr) | NA | 1925 | NA | 1.18(1.03 - 1.37) | NA |
| Yan Luo | 2024 | China | RCS | 910 | 49.60% | 65.98 | TyG index | 1-1.2 (yr) | AF-RFA | NA | 189 | NA | 1.47(1.16–1.87) |
| Caravaca | 2025 | Spain | PCS | 2,902 | 47.50% | 77.00 | TyG index | 2 (yr) | NA | NA | 208 | NA | 1.82(1.15–2.89) |
| Aobo Gong | 2025 | China | RCS | 864 | 55.30% | 67.69 | TyG index | 3.9 (yr) | NA | NA | NA | NA | 1.77(1.44–2.17) |
| Yonggu Lee | 2020 | Korea | PCS | 8,175 | 46.70% | 51.50 | HOMA-IR | 11.5(yr) | NA | 136 | NA | 1.61(1.14 - 2.29) | NA |
| Yang Ling | 2022 | China | RCS | 549 | 80.20% | 63.00 | TyG index | 2.9 (yr) | NA | 42 | NA | 1.58(1.27 - 1.97) | NA |
| Hao Huang | 2025 | UK | PCS | 360,953 | 45.10% | 56.30 | eGDR | 13.8 (yr) | NA | 23,638 | NA | 1.18(0.78 - 1.58) | NA |
| Shanshan Shi | 2024 | UK | PCS | 409,705 | 45.70% | 56.40 | TyG index | 13.9 (yr) | NA | 26,092 | NA | 1.32(1.17 - 1.87) | NA |
| Fontes | 2012 | USA | PCS | 3,023 | 45.20% | 59.20 | HOMA-IR | 10 (yr) | NA | 279 | NA | 1.18(0.84 - 1.65) | NA |
| Tang | 2022 | China | RCS | 275 | 69.40% | 57.30 | TyG index | 2.2 (yr) | AF-RFA | NA | 70 | NA | 2.02(1.41–4.12) |
| Li XZ | 2024 | China | RCS | 899 | 41.90% | 64.45 | eGDR | 0.9 (yr) | AF-RFA | NA | 296 | NA | 1.08(0.5 - 1.87) |
| Kan | 2024 | China | RCS | 325 | 60.90% | 60.60 | TyG index | 1 (yr) | AF-RFA | NA | 79 | NA | 3.39(2.08–5.53) |
| Johnson | 2015 | Japan | RCS | 1,414 | 77.00% | 62.00 | HOMA-IR | 0.8-3.2 (yr) | AF-RFA | NA | 320 | NA | 1.57(1.08–2.28) |
| Tze-Fan Chao | 2013 | China (Taiwan) | RCS | 122,524 | 73.70% | 50.60 | HOMA-IR | 6.3 (yr) | NA | 2,339 | NA | 1.19(1.01 - 1.59) | NA |
| Qing Yan | 2024 | China | RCS | 375 | 64.30% | 63.20 | TyG index | 1 (yr) | AF-RFA | NA | 67 | NA | 1.45(0.78 - 3.78) |
| Naoko Hijioka | 2018 | Japan | PCS | 114 | 78.00% | 62.60 | HOMA-IR | 1 (yr) | AF-RFA | NA | 28 | NA | 1.29(1.08–1.53) |
| Jingwei Zhang | 2023 | China | RCS | 424 | 70.80% | 58.20 | TyG index | 2.8 (yr) | AF-RFA | NA | 117 | NA | 2.02(1.37–3.25) |
| Zhen Tan | 2025 | UK | PCS | 31,733 | 57.28% | 59.19 | eGDR | 12.8 (yr) | NA | 3892 | NA | 1.54(1.31 - 1.76) | NA |
| Aiko Takami | 2025 | Japan | RCS | 818 | 70.70% | 67.00 | HOMA-IR | 2 (yr) | AF-RFA | NA | 208 | NA | 2.62(1.44–4.80) |
| Pil-Sung Yang | 2016 | Korea | PCS | 142 | 78.20% | 63.30 | HOMA-IR | 0.8-3.2 (yr) | AF-RFA | NA | 30 | NA | 1.14(0.38 - 1.69) |
| Xinyi Yu | 2025 | China | PCS | 11,663 | 49.20% | 67.00 | TyG index | 11.1 (yr) | NA | 1343 | NA | 1.3(1.03 - 1.67) | NA |
| Zhe Wang | 2022 | China | PCS | 384 | 61.50% | 58.10 | HOMA-IR | 1.2 (yr) | AF-RFA | NA | 90 | NA | 1.26(1.1–1.46) |
| Jianliang Liu | 2025 | China | RCS | 293 | 44.70% | 60.00 | TyG index | 1.4-3 (yr) | NA | NA | 88 | NA | 1.47(1.01–2.14) |
| Johnson | 2018 | Sweden | PCS | 772 | 43.00% | 64.50 | HOMA-IR | 10.4 (yr) | AF-RFA | 72 | NA | 1.55(1.35 - 1.82) | NA |
| Muhammad | 2023 | Sweden | PCS | 32,917 | 50.00% | 58.00 | TyG index | 20 (yr) | AF-RFA | 6950 | NA | 1.16(0.89 - 1.04) | NA |
| Jung-Chi Hsu | 2023 | China (Taiwan) | RCS | 28,618 | 46.90% | 64.50 | HOMA-IR | 4 (yr) | NA | 1189 | NA | 1.24(1.11 - 1.39) | NA |

Abbreviations:TyG index=Triglyceride-glucose index;eGDR=estimated glucose disposal rate;HOMA-IR=Homeostasis Model Assessment of Insulin Resistance;AF-RFA=Radiofrequency ablation for atrial fibrillation;RCS=retrospective cohort study; PCS=prospective cohort study.
