# Supplementary material for: Insulin resistance and atrial fibrillation: from disease onset to post-ablation outcomes: a systematic review and meta-analysis
Source: Front Cardiovasc Med. 2026 Jan 8;12:1700730. doi: 10.3389/fcvm.2025.1700730 (PMC12823920; doi:10.3389/fcvm.2025.1700730)
Supplement: Supplementary file 4 [file Table4.docx]

Supplementary Table 3

| Author/Year | Bias due to confounding | Bias in selection of participants into the study | Bias in classification of interventions | Bias due to deviations from intended interventions | Bias due to missing data | Bias in measurement of outcomes | Bias in selection of the reported result | Overall risk of bias |
| --- | --- | --- | --- | --- | --- | --- | --- | --- |
| Yongwei Huang 2024 | Moderate | Moderate | Low | Moderate | Low | Low | Low | Moderate |
| Zhe Wang 2024 | Moderate | Low | Low | Low | Low | Low | Low | Moderate |
| Zhihong Zuo 2025 | Moderate | Low | Low | Low | Low | Low | Low | Moderate |
| Sixiang Jia 2024 | Moderate | Low | Low | Low | Low | Low | Low | Moderate |
| Xiao Liu 2023 | Low | Low | Low | Low | Low | Low | Low | Low |
| Yan Luo 2024 | Moderate | Low | Low | Low | Low | Low | Low | Moderate |
| Caravaca 2025 | Moderate | Low | Low | Low | Low | Low | Low | Moderate |
| Aobo Gong 2025 | Moderate | Low | Low | Low | Low | Low | Low | Moderate |
| Yonggu Lee 2020 | Moderate | Low | Low | Low | Low | Low | Low | Moderate |
| Yang Ling 2022 | Moderate | Low | Low | Low | Low | Low | Low | Moderate |
| Hao Huang 2025 | Moderate | Low | Low | Low | Low | Low | Low | Moderate |
| Shanshan Shi 2024 | Moderate | Low | Low | Low | Low | Low | Low | Moderate |
| Fontes 2012 | Moderate | Low | Low | Low | Moderate | Low | Low | Moderate |
| Tang 2022 | Moderate | Low | Low | Moderate | Moderate | Low | Low | Moderate |
| Li XZ 2024 | Moderate | Moderate | Low | Low | Low | Low | Low | Moderate |
| Kan2024 | Moderate | Low | Low | Low | Low | Low | Low | Moderate |
| Johnson2015 | Moderate | Low | Low | Low | Low | Low | Low | Moderate |
| Tze-Fan Chao 2013 | Moderate | Low | Low | Low | Low | Low | Low | Moderate |
| Qing Yan 2024 | Moderate | Low | Low | Low | Low | Low | Low | Moderate |
| Naoko Hijioka 2018 | Moderate | Low | Low | Low | Low | Low | Low | Moderate |
| Jingwei Zhang 2023 | Moderate | Low | Low | Low | Low | Low | Low | Moderate |
| Zhen Tan 2025 | Moderate | Low | Low | Low | Low | Low | Low | Moderate |
| Aiko Takami 2025 | Moderate | Low | Low | Low | Low | Low | Low | Moderate |
| Pil-Sung Yang 2016 | Moderate | Low | Low | Low | Low | Low | Low | Moderate |
| Xinyi Yu 2025 | Moderate | Low | Low | Low | Low | Low | Low | Moderate |
| Zhe Wang 2022 | Moderate | Low | Low | Low | Low | Low | Low | Moderate |
| Jianliang Liu 2025 | Moderate | Low | Low | Low | Low | Low | Low | Moderate |
| Johnson 2018 | Moderate | Low | Low | Low | Low | Low | Low | Moderate |
| Muhammad 2023 | Low | Low | Low | Low | Low | Low | Low | Low |
| Jung-Chi Hsu 2023 | Low | Low | Low | Low | Low | Low | Low | Low |
